# Proteomic Analysis of *Fusarium solani* Isolated from the Asian Longhorned Beetle, *Anoplophora glabripennis*

**DOI:** 10.1371/journal.pone.0032990

**Published:** 2012-04-09

**Authors:** Erin D. Scully, Kelli Hoover, John Carlson, Ming Tien, Scott M. Geib

**Affiliations:** 1 Intercollege Program in Genetics, The Huck Institutes of the Life Sciences, The Pennsylvania State University, University Park, Pennsylvania, United States of America; 2 Department of Entomology, Center for Chemical Ecology, The Pennsylvania State University, University Park, Pennsylvania, United States of America; 3 School of Forest Resources, The Pennsylvania State University, University Park, Pennsylvania, United States of America; 4 Department of Bioenergy Science and Technology (World Class University), Chonnam National University, Buk-Gu, Gwangju, Korea; 5 Department of Biochemistry and Molecular Biology, The Pennsylvania State University, University Park, Pennsylvania, United States of America; 6 Tropical Crop and Commodity Protection Research Unit, USDA-ARS Pacific Basin Agricultural Research Center, Hilo, Hawaii, United States of America; Oregon State University, United States of America

## Abstract

Wood is a highly intractable food source, yet many insects successfully colonize and thrive in this challenging niche. Overcoming the lignin barrier of wood is a key challenge in nutrient acquisition, but full depolymerization of intact lignin polymers has only been conclusively demonstrated in fungi and is not known to occur by enzymes produced by insects or bacteria. Previous research validated that lignocellulose and hemicellulose degradation occur within the gut of the wood boring insect, *Anoplophora glabripennis* (Asian longhorned beetle), and that a fungal species, *Fusarium solani* (ATCC MYA 4552), is consistently associated with the larval stage. While the nature of this relationship is unresolved, we sought to assess this fungal isolate's ability to degrade lignocellulose and cell wall polysaccharides and to extract nutrients from woody tissue. This gut-derived fungal isolate was inoculated onto a wood-based substrate and shotgun proteomics using Multidimensional Protein Identification Technology (MudPIT) was employed to identify 400 expressed proteins. Through this approach, we detected proteins responsible for plant cell wall polysaccharide degradation, including proteins belonging to 28 glycosyl hydrolase families and several cutinases, esterases, lipases, pectate lyases, and polysaccharide deacetylases. Proteinases with broad substrate specificities and ureases were observed, indicating that this isolate has the capability to digest plant cell wall proteins and recycle nitrogenous waste under periods of nutrient limitation. Additionally, several laccases, peroxidases, and enzymes involved in extracellular hydrogen peroxide production previously implicated in lignin depolymerization were detected. *In vitro* biochemical assays were conducted to corroborate MudPIT results and confirmed that cellulases, glycosyl hydrolases, xylanases, laccases, and Mn- independent peroxidases were active in culture; however, lignin- and Mn- dependent peroxidase activities were not detected While little is known about the role of filamentous fungi and their associations with insects, these findings suggest that this isolate has the endogenous potential to degrade lignocellulose and extract nutrients from woody tissue.

## Introduction

Most beetles in the family Cerambycidae develop deep in woody tissues where access to sugar monomers present in plant cell wall polysaccharides is impeded by the presence of a recalcitrant lignin barrier and other essential nutritional resources, including proteins, lipids, sterols, and vitamins, are deficient or absent altogether [1]. Many cerambycids that thrive in this suboptimal environment overcome these barriers by preferentially targeting weakened or stressed trees, whose woody, intractable components have been pre-digested by wood-degrading fungi, which degrade lignocellulose into easily digestible mono- and di- saccharides and synthesize other essential dietary components [2]. Alternatively, other cerambycids harbor external wood-degrading fungi, which are physically inoculated into host trees; the fungi colonize the larval tunnels, digest carbon polymers, and serve nutrient provisioning roles for the insect or fungal enzymes are ingested by the insect to aid in lignocellulose digestion in the gut [3]. Unlike other cerambycids, the Asian longhorned beetle (*Anoplophora glabripennis*, Coleoptera: Cerambycidae), an exotic insect native to China first detected in the U.S. in the early 1990's, attacks both weakened [4] and healthy [5] deciduous trees in the absence of external wood-degrading fungi. This beetle also enjoys a broad host range, which includes over 21 deciduous tree species [6], [7].

*A. glabripennis* larvae face a number of challenges acquiring nutrients as they grow and develop deep in the sapwood (and heartwood in some tree species) where the lignin: nitrogen ratio is high. Woody tissue is primarily composed of three polymeric materials: cellulose, hemicellulose and lignin. Cellulose is a linear polymer of glucose linked by β-1,4glycosidic bonds, accounting for approximately 45% of wood by weight. Its linear structure and extensive hydrogen bonding increases crystallinity of the macromolecule and decreases accessibility of hydrolytic enzymes [8]. Hemicellulose accounts for approximately 15 to 35% of wood by weight depending on tree species and is also bound by β-1,4 linkages. Unlike cellulose, hemicellulose has much greater structural heterogeneity and is primarily comprised of xylose chains forming a xylan structure in secondary cell walls of hardwood tree species. In addition, other lesser abundant monomers typically found in hemicellulose include galactose, rhamnose, arabinose, mannose and their acid derivatives [9]. Lignin is an amorphous structural aromatic polymer, which is often esterified to uronic residues present in hemicellulose and cross-linked to cellulose through ether and glycosidic linkages, protecting these polysaccharides from hydrolytic enzymes [10]. Furthermore, some evidence indicates that cell wall proteins present in xylem elements are cross-linked with lignin and other cell wall polysaccharides, protecting them from proteolysis, suggesting that lignocellulose and hemicellulose degradation may be required for protein acquisition [11]. Phenylpropanoid units, including coumaryl, coniferyl, and sinapyl alcohol, are the precursors of lignin. Oxidation of these phenols yields free radicals that undergo radical coupling to form a polymer comprised of over 12 types of chemical linkages and dominated by C-C and ether linkages, which are invulnerable to hydrolysis and can only be broken through radical oxidative depolymerization [12]. Its lack of stereoregularity and periodicity and its condensed, insoluble properties make lignin resistant to most forms of enzymatic attack [13].

Current methods of degradation of lignin have been described for white rot basidiomycete fungi. These fungal species utilize secreted heme peroxidases with high redox potentials, including lignin-, Mn-, and versatile- peroxidases, to depolymerize the lignin molecule through oxidization of non-phenolic C-C and ether linkages using hydrogen peroxide as an oxidant [14]. Laccases, or multicopper phenol oxidases, are also produced by some lignin-degrading basidiomycetes, which solely oxidize the phenolic hydroxyl groups that comprise less than 10% of the total linkages in an intact lignin biopolymer. Although these enzymes can indirectly degrade more recalcitrant linkages in the presence of synthetic redox mediators, such as the diammonium salt of 2,2′-azine-*bis* (3-ethylbenzothiazoline-6-sulfonic acid) (ABTS), natural varieties of these mediators have yet to be discovered; thus, the full biochemical role of laccases in natural lignin depolymerization processes remain obscure [15]. In brown rot basidiomycetes, hydroxy radicals generated through Fenton reactions initiated by iron III reductase, catalyzed by heme domains in cellobiose dehydrogeases, are responsible for lignin modification [10]. Although these reactive hydroxy radicals induce only partial lignin depolymerization, many speculate that Fenton reactions occur in tandem with radical oxidative processes to expedite complete lignin depolymerization since Fenton-type lignin metabolites have been detected in association with white-rot mediated lignin degradation [16]. Outside the basidiomycetes, the process of lignin degradation by other types of fungi is largely unknown; while some bacteria can degrade aromatic monolignols, dilignols, and other phenolic linkages found in lignin, only partial lignin depolymerization and modification have been documented in bacteria. Thus, bacterial lignin degradation is not well understood and highly speculative [17], [18], [19], [20], [21].

From previous studies, we demonstrated that lignin, cellulose, and hemicellulose degradation occur in the guts of larval *A. glabripennis* [22], [23], [24]. Although many insects, including cerambycids, can produce endogenous cellulases and other cell wall degrading enzymes [25], [26], [27], [28], [29], [30], [31] as well as enzymes capable of oxidizing phenolic linkages present in lignin [12], [25], [26], [27], [28], [29], [30], [32], [33], [34], there is no conclusive evidence that insects endogenously produce enzymes capable of oxidizing the predominant C-C or ether linkages required for full lignin depolymerization, which are definitively broken in the *A. glabripennis* gut [22]. However, *A. glabripennis* harbors gut microbiota that likely contribute to digestive physiology and help this insect overcome barriers associated with extracting nutrients from woody tissue, including radical oxidation of non-hydrolyzable bonds in lignin. Previous 16 s rRNA-based metagenomic analyses of the gut bacterial communities of larvae reared in several different suitable host tree species revealed a community dominated by Actinobacteria and Proteobacteria that appears to display a tremendous degree of plasticity at lower taxonomic ranks. For example, striking shifts in alpha diversity and taxonomic composition were observed in insects reared in different host tree species with little impact on larval fitness or ability to degrade cellulose and xylan [23]. In contrast, the fungal community was relatively static in comparison and was consistently dominated by a single fungal taxon regardless of collection site or host tree species, demonstrating a stable relationship between this fungal isolate and *A. glabripennis*. Multilocus phylogenetic analysis encompassing the ITS, EF, and LSU loci convincingly places this isolate in the *Fusarium solani* species complex [35].

Despite their status as notorious plant and animal pathogens, *Fusarium spp.* are occasionally found in non-pathogenic associations with beetles [36]. One of the most well studied examples is the relationship between Ambrosia beetles (Coleoptera, Curculionidae) and *Fusarium solani*. Ambrosia beetles harbor fungi in special external structures called mycangia, inoculating the fungus into the tree to digest lignocelluose and synthesize other nutrients, including ergosterols required for pupation [37]. However, in many cases, the nature of the relationship between beetles and their respective *Fusarium* affiliates is unclear; although the presence of the fungus appears to directly improve insect growth and fecundity, their precise contributions to insect physiology and biochemistry are largely unknown [36]. While we are just beginning to understand the relationship between the Asian longhorned beetle and its gut-associated *F. solani* isolate, it is known that *A. glabripennis* larval galleries are typically visually free of fungal inoculum indicating that if this isolate is contributing to lignocellulose digestion or other physiological processes *in vivo*, these processes occur within the gut rather than in the external environment [35].

Furthermore, it is known that *F. solani* isolates comprise a metabolically versatile species complex that can colonize many diverse niches and persist in extreme environments. Owing to this versatility, these fungi can often extract glucose from exotic carbon sources, including pyrene and benzopyrene [38], [39] and often produce an impressive arsenal of xenbiotic degrading enzymes capable of degrading many common aromatic hydrocarbon pollutants, including chlorobenzenes, polychlorinated biphenyls, and phenanthrenes [40]. One of the hallmark characteristics of lignin degrading enzymes is their lack of substrate specificity [41], leading many to speculate that the enzymes involved in xenobiotic oxidoreductive processes may also oxidize recalcitrant bonds present in lignin. Early wood block surveys conducted with freshwater *Nectria* isolates (anamorph: *Fusarium*) were initially discouraging as these isolates did not induce substantial weight loss characteristic of soft rot processes, despite prolific production of cell wall polysaccharide degrading enzymes and phenol oxidases [42], [43]. However, studies on other *F. solani* isolates convincingly demonstrated their abilities to oxidize aromatic rings and side chains of synthetic lignin compounds, efficiently metabolize both Kraft and Klason lignin, and thrive on lignocellulose-based substrates as sole sources of carbon [44], [45], [46], [47]. Notably, maximal evolution of ^14^CO_2_ from radiolabelled lignin rings and side chains occurred substantially earlier in *F. solani* isolates in comparison to white rot basidiomycetes and some *Fusarium* isolates degraded both lignin and polysaccharides simultaneously [46], clearly indicating a strong lignin-degrading propensity and suggesting that these fungi may harbor highly efficient lignin degrading enzymes that could be used to enhance industrial cellulosic ethanol production. Additionally, the recent genome sequence of *Nectria haematococca* (anamorph: *F. solani*; mating population VI (MPVI)) also includes a putative lignin peroxidase ortholog (protein ID 4582) [48]; to our knowledge, its activity has not been verified *in vitro*, but this protein possesses all of the functional domains required for lignin peroxidase activity.

Due to its strong association with *A. glabripennis* and its potential metabolic versatility and lignocellulose degrading properties, the goal of this study was to survey and characterize the lignocellulolytic, cell wall polysaccharide degrading, and other nutrient extracting capacities of this *F. solani* isolate using *de novo* peptide sequencing and *in vitro* biochemical assays of extracellular proteins produced by the fungus grown on a wood-based substrate.

## Materials and Methods

### Source of larval *A. glabripennis* associated *F. solani* culture

Fungus cultures were obtained from *A. glabripennis* larvae maintained at Penn State University, Department of Entomology, University Park, PA USA. Adult *A. glabripennis* maintained in a quarantined greenhouse were allowed to oviposit into potted sugar maple (*Acer saccharum*) trees, which are highly preferred hosts of these beetles [7]. Subsequently, eggs were allowed to hatch and larvae were permitted to mature in the trees' woody tissues. After a period of 90 days, healthy larvae feeding on inner wood were collected and fungi were cultured from larval guts as described previously [22] to create single spore cultures. This isolate is currently curated at the American Type Culture Collection under the accession number MYA 4552.

### Solid wood substrate fungal cultures and fungal enzyme extraction

Several agar plugs from a culture of *F. solani* (MYA 4552) described above were used to inoculate a solid wood substrate in polypropylene growth bags (Unicorn, Commerce, TX, USA) containing 250 g red oak wood chips, 30 g millet, 15 g wheat bran, and 400 mL distilled water at 30°C [49]. Previous studies in *Phanerochaete chrysosporium* revealed that white rot fungi require easily metabolizable carbohydrates to induce production of peroxidases involved in lignin degradation as this process typically occurs during periods of secondary metabolism and under conditions of extreme nitrogen limitation [10]. Although there is limited evidence that certain *Fusarium solani* strains can thrive on lignin as a sole source of carbon and do not require easily metabolizable carbohydrates, including cellulose, glucose, or soluble starches, to induce lignin degrading enzymes [47], other strains do require these components and will not colonize lignocelluose-based substrates in their absence. Thus, general growth conditions required for induction of lignocellulolytic genes in *Fusarium* are poorly understood and abilities to colonize lignocellulose substrates vary tremendously within this species complex [50]. To promote initial substrate colonization regardless of inherent metabolic potential, the medium was augmented with millet and the culture was grown for an extended time period to expend tractable carbohydrate and protein resources present in the growth medium and to induce physiological processes characteristic of secondary metabolism.

Approximately one month after inoculation, total fungal enzymes were extracted from the entire culture as previously described [49] by mixing bag contents with one volume of 0.5 M NaCl and incubating for 2 h at 4°C with constant stirring. The mixture was then squeezed through cheesecloth and centrifuged at 15,000×*g* for 30 min at 4°C to remove cellular debris/wood and preferentially purify proteins secreted into the extracellular environment. Ammonium sulfate was added to the filtrate over a 30 minute time period until the solution reached 100% saturation to precipitate proteins and to preserve proteins in their native conformation. The suspension was incubated overnight at 4°C with stirring and the preparation was centrifuged at 15,000×*g* for 30 min at 4°C. The protein pellets were dissolved in 50 ml of water and trace amounts of ammonium sulfate were removed by repeated concentration (Amicon, 10-kDa cutoff) and re-suspension in 50 ml of water. The final extractions were partitioned into 1 ml aliquots and stored at −80°C.

### MudPIT analysis

One mg of total *F. solani* protein extract was digested with trypsin in solution [51]. Briefly, the protein sample was lyophilized and resuspended in 100 µl of 6 M urea, 100 mM Tris buffer (pH 7.8). Denatured proteins were reduced by adding 5 µl of 200 mM dithiothreitol (DTT) in 100 mM Tris (pH 7.8) and incubating for 1 h at room temperature. The protein sample was subsequently alkylated by adding 20 µl of 200 mM iodoacetamide in 100 mM Tris (pH 7.8) and incubating for another hour at room temperature. Residual iodoacetamide was then consumed by adding another 20 µl of 200 mM DTT in 100 mM Tris (pH 7.8) followed by a1 hour incubation at room temperature. The sample was diluted with water to a working volume of 0.9 ml; then, 20 µg of trypsin in 0.1 mL of water was added to the sample, (Trypsin Gold, Promega Corporation, Madison WI) bringing the final reaction volume to 1 ml. The protein sample was then completely digested overnight at 37°C and the reaction was halted the following day by lowering the pH to <6.0 with acetic acid. Residual salts were removed from the sample through repeated concentration in a SpeedVac and resuspension in water.

Multidimensional Protein Identification Technology (MudPIT) analysis was performed at the Penn State Hershey Medical Center Mass Spectrometry Core Research Facility. Two-dimensional liquid chromatography was performed to highly fractionate the sample. In brief, a strong cation exchange (SCX) column was used to separate the protein extract into 15 fractions. These fractions were subsequently separated on a C-18 column and each sub-fraction was directly spotted onto a MALDI target plate (370 spots/fraction). A total of 5500 MALDI spots were prepared in this manner. Next, tandem MS was performed for each spot on an ABI 4800 MALDI-TOF-TOF (Applied Biosystems, Foster City, CA, US) to determine *de novo* peptide sequences. These fragments were mapped to the *Nectria haematococca/F. solani* Mating Population VI (MPVI) reference protein set (http://genome.jgi-psf.org/Necha2/Necha2.home.html) [48] using ProteinPilot 3.0 Software's Paragon Algorithm (Applied Biosystems, Foster City, CA, US). This genome was chosen due to its phylogenetic proximity to the *A. glabripennis*-derived *F. solani* isolate [35], and it was the only suitable reference genome within the *F. solani* species complex that was publicly available at the time of study. In this analysis, the reference proteome is digested *in silico* and *de novo* peptide sequences determined by tandem MS are mapped back to annotated peptides for identification and scored based on mapping quality and coverage. Mapping quality is determined by sequence similarity at the amino acid level and the number of unique mapping locations in the reference proteome; thus, peptides with high amino acid similarity to their predicted reference proteins and peptides that map uniquely to a single protein in the reference proteome are given a higher score. Coverage is determined by the number of unique peptides that map to a single reference protein and proteins covered by multiple peptides are scored more favorably. These parameters are integrated into the ‘unused’ score, which is used to infer the confidence of a protein match. In this analysis, an unused score of 1.3 represents a significant protein match at 95% confidence; significant protein matches were annotated using the *Nectria haematococca/F. solani* Mating Population VI (MPVI) 2.0 genome database (abbreviated Necha 2.0).

KEGG, gene ontology (GO), and InterPro (IPR) annotations present in genome database were applied to proteins detected by MudPIT analysis. In addition, the full *N. haematococca* MPVI amino acid sequence for each protein identified was analyzed for the presence of signal peptides using both neural network and Hidden Markov model (HMM) methodologies using the SignalP 3.0 web server [52]. For neural network analysis, the mean S score was used to determine presence of a signal peptide and HMM predictions were based on the C_max_ score. In an attempt to infer the function of proteins detected in the secretome that were annotated as ‘hypothetical’ in the *N. haematococca* database, the peptide sequences were extracted from the reference genome and were compared to the non-redundant protein database using blastp (blast 2.2.26+). From these annotations, we summarized protein classes present, with a focus on enzymes involved in plant cell wall digestion and protein acquisition. Additionally, to confirm that proteins detected in the MudPIT data were enzymatically active in culture we performed *in vitro* cellulase, xylanase, lignin peroxidase, manganese-dependent and –independent peroxidase assays.

### *In vitro* cellulase and xylanase activities

*In vitro* cellulase, glycoside hydrolases and xylanase activities of fungal enzyme extracts were confirmed by measuring release of reducing sugars from cellulose or xylan-based substrates using the dinitrosalicylic acid (DNS) assay [53], [54]. Protein concentration was measured using Bradford chemistry [55], [56] with BSA as a protein standard (0–20 µg); samples were diluted to a working concentration of approximately 60 µg/ml in sodium citrate buffer (50 mM, pH 5.5). To assay for ability to degrade cellulose, we quantified release of reducing sugars from cellulose-based substrates, including microcrystalline cellulose (Avicel) and carboxymethyl cellulose (CMC). To quantify glycoside hydrolase activity directed at β-1,4 linkages present in D-glycopyranosyl containing compounds, salicin, a β-1,4 conjugated phenolic glycoside, was utilized. For CMC and salicin DNS assays, 500 µl of a 2% substrate solution (in 50 mM sodium citrate buffer, pH 5.5) was combined with 30 µg (500 µl of 60 µg/ml) of fungal extract; however, due to substrate insolubility, a 1% solution of microcrystalline cellulose was substituted. For xylanase activity, 500 µl of a 1% xylan solution (in 50 mM sodium citrate buffer, ph 5.5, xylan from birch wood, Sigma Aldrich Corporation) was combined with 30 µg (500 µl of 60 µg/ml dilution) of fungal extract. Three technical replicates were performed and non-enzyme controls were run to detect background release of reducing sugars from cellulose and xylan based substrates. For all assays, 100 µl of the reaction mixture was removed at time 0 and read at 540 nm to allow for subtraction of background reducing sugars. Reactions were incubated at 37°C for 120 min and 100 µl aliquots were removed from each reaction after 60 and 120 minutes to record release of reducing sugars over time. For each aliquot, 100 µl DNS reagent was added to halt enzyme activity [54] and samples were boiled for 8 min in a water bath. 150 µL aliquots of DNSA reaction were read at 540 nm on a SpectraMax™ microplate reader (Molecular Devices Corp.) and were compared with a glucose standard curve (20 to 1000 µg) to quantify concentration of reducing sugars at each time point.

### Zymogram analysis

SDS-PAGE gels [57] were performed with modifications to independently verify cellulase or xylanase activity through zymogram techniques [58], [59], [60]. Twelve percent acrylamide separation gels were prepared containing either 0.1% carboxymethyl cellulose or 0.1% xylan from birch wood. Fungal enzyme extracts prepared as described above were loaded onto each gel in duplicate (20 µg protein/lane) with a pre-stained protein standard (SeeBlue Plus, Invitrogen, Carlsbad, CA), so that the gel could be cut vertically in half after electrophoresis to produce two identical acrylamide gels. The first half of the gel was stained with colloidal blue to visualize proteins as a reference and imaged using a densitometer (GS-800, Bio-Rad, Hercules, CA). The second half was used for zymogram analysis. Zymogram gels were rinsed in sodium citrate buffer (50 mM, pH 5.5) containing 1% Triton X-100 for 1 h at room temperature to remove SDS [58]. This was followed by 1.5 h incubation in sodium citrate buffer (50 mM, pH 5.5) to allow for enzyme activity against the substrates. At this point, gels were stained with 0.1% Congo red for 30 min and destained in 1 M NaCl to reveal zones of clearing indicative of degradation of polysaccharide substrates. Gels were imaged under ultraviolet light to highlight zones of clearing and aligned with colloidal blue stained gels using the pre-stained protein standard as a reference.

### *In vitro* lignin peroxidase, manganese peroxidase, and laccase activities

Ligninolytic activity of the fungal extract was further assessed through *in vitro* approaches. Lignin peroxidase activity was assayed by the oxidation of veratryl alcohol to veratraldehyde as an increase in A_310_ [41]. Approximately 50 µg of protein were added to 1 mL of a solution containing 25 mM sodium tartrate (pH 3.0) and 20 mM veratryl alcohol. The reaction was initiated by addition of H_2_O_2_ at a concentration of 2 mM and the absorbance at A_310_ was recorded. Mn-dependent and -independent peroxidase activities were measured by the oxidation of 2,6-dimethoxyphenol as an increase in A_470_ [61]. Approximately 50 µg of protein extract were added to a solution containing 20 mM 2,6-dimethoxyphenol and 0.5 M sodium tartrate (pH 4.5), either with or without 20 mM manganese sulfate (for Mn-dependent and -independent activity, respectively). Total reaction volume was 1 ml and the peroxidase reaction was initiated by addition of H_2_O_2_ to obtain a final concentration of 2 mM. Absorbance at 470 nm was recorded. Laccase activity was verified using 2,6-dimethoxyphenol as a substrate [62]. Approximately 50 µg of protein extract were added to a solution containing 20 mM 2,6-dimethoxyphenoland 0.5 M sodium tartrate (pH 4.5) to a total volume of 1 ml. Absorbance was then read at 470 nm. All assays were performed in triplicate and mean change in absorbance versus a no enzyme control over five minutes was recorded and this difference was used as a measure of enzyme activity. One unit of activity was defined as the amount of enzyme that oxidizes 1 µmol of substrate per minute.

### Non-denaturing PAGE and heme staining

All known peroxidases with deconstructive activity directed at intact lignin polymers are extracellular heme oxides containing a heme prosthetic group [10], [60], [63]. In an attempt to detect extracellular proteins present containing this vital prosthetic group, heme stained gels were prepared. In brief, non-denaturing polyacrylamide gel electrophoresis (native PAGE) was run following the methods of Laemmli [57] with a gel containing 12% acrylamide and lacking SDS in the gel and running buffer. To further preserve the functional integrity of the proteins, the samples were not boiled prior to loading on the gel. Fungal enzyme extracts were loaded onto the gel in duplicate (20 µg protein/lane) with protein standards so that the gel could be cut vertically in half after electrophoresis to produce two identical acrylamide gels. The first half of the gel was stained with colloidal blue to visualize proteins as a reference and imaged with a densitometer (GS-800, Bio-Rad, Hercules, CA). The second half of the gel was stained to identify proteins containing a heme prosthetic group. In brief, the second half of the gel was incubated in a Tris-MeOH solution (20 mM Tris-HCl, pH 7.3, 50% MeOH) for 30 min, followed by a 45-min incubation in heme stain solution (25 mM acetate buffer, pH 5.3, 0.25% benzidine HCl, 25% MeOH, and 0.75% H_2_O_2_). The gel was then rinsed in 25% MeOH and stored in 0.1 M Tris-HCl, pH 7.3. Heme containing proteins stained dark brown and were matched with colloidal blue stained proteins on the reference gel. Although other non-lignin degrading proteins that contain a heme prosthetic group will stain (cytochromes) using this approach, these proteins are primarily intracellular and are found in association with mitochondria.

## Results

### MudPIT Analysis

A total of 8400 spectra were collected from tandem MS and, of these, 4740 spectra were utilized to identify 3638 unique peptides at 95% confidence (Table 1). These 3638 peptides reliably mapped to 398 distinct proteins in the *N. haemotococca* reference genome (Table 1). Full protein scores for all proteins detected are presented in the supplemental information (Table S1). By using the full amino acid sequence from the *N. haematococca* genome, 175 proteins were found to have a signal peptide using SignalP 3.0 neural network model and 183 using HMM model (Table S2). Over half of the proteins (224 for neural network and 203 for HMM) did not contain a detectable signal peptide probably due to incidental lysis of cell membranes during the protein extraction or errors in computational signal peptide prediction. Through functional annotation, InterPro information was applied to 309 of these proteins, GO IDs to 275 proteins, and KEGG annotations to 128 proteins. Full InterPro, GO, and KEGG annotation are provided in supplemental information (Tables S3, S4, and S5 respectively). Furthermore, there were 20 InterPro IDs that had 5 or more proteins classified to them (Table 2). Many of these abundant Interpro IDs are related to cellulose/carbohydrate binding and degradation, protein/peptide degradation, and general metabolic functions. Focusing more closely on proteins that hydrolyze sugars, an analysis of glycoside hydrolase (GH) families was performed using the InterPro IDs and blast searching the reference proteins against the NCBI non-redundant database.

**Table 1:** MudPIT summary data.

Unused (% Conf) Cutoff	Proteins Detected	Distinct Peptides	Spectra Identified	% Total Spectra Used
>2.0 (99)	264	3219	4279	51
>1.3 (95)	398	3638	4740	56.4

**Table 2:** Most Abundant InterPro IDs Identified in MudPIT analysis.

InterPro ID	# Proteins with annotation	InterPro Description
IPR000379	16	Esterase/lipase/thioesterase
IPR001138	14	Fungal transcriptional regulatory protein, N-terminal
IPR000254	8	Cellulose-binding region, fungal
IPR001764	8	Glycoside hydrolase, family 3, N-terminal
IPR000209	8	Peptidase S8 and S53, subtilisin, kexin, sedolisin
IPR007219	7	Fungal specific transcription factor
IPR002772	7	Glycoside hydrolase, family 3, C-terminal
IPR003439	6	ABC transporter
IPR001410	6	DEAD/DEAH box helicase
IPR001650	6	Helicase, C-terminal
IPR007484	6	Peptidase M28
IPR003137	6	Protease-associated PA
IPR003593	5	AAA ATPase
IPR001757	5	ATPase, E1–E2 type
IPR006045	5	Cupin
IPR008250	5	E1–E2 ATPase-associated region
IPR005834	5	Haloacid dehalogenase-like hydrolase
IPR000719	5	Protein kinase
IPR010259	5	Proteinase inhibitor I9, subtilisin propeptide
IPR002290	5	Serine/threonine protein kinase

In total, 48 different proteins classified into 28 GH families were represented in the protein extract, encompassing ∼8% of all proteins identified (Table 3). The most abundant GH was GH 3, which are proteins represented by a broad range of enzyme classes, including β-glucosidase (EC 3.2.1.21), xylan-1,4- β-xylosidase (EC 3.2.1.37), β-N-actetylhexosamidase (EC 3.2.1.52), and α-L-arabinofuranosidase (EC 3.2.1.55). Specifically β-glucosidase (EC 3.2.1.21) was identified in our sample from this GH family through cross-annotation with the KEGG database (Table S5). In addition, α-amylase (EC 3.2.1.1; GH 13), glucoamylase (EC 3.2.1.3; GH 15), licheninase (EC 3.2.1.73; GH 16), glucan 1,3-β-glucosidase (EC 3.2.1.58; GH 17), chitinase (EC 3.2.1.14; GH 28), β-hexosaminidase (EC 3.2.1.52; GH 20), α-glucosidase (EC 3.2.1.20; GH 37), β-galactosidase (EC 3.2.1.23; GH 35), and α,α-trehalase (EC 3.2.1.28; GH 37) were identified in the proteome. A full description of potential enzymes in other GH families can be found at www.cazy.org [63]. We were unable to annotate other GH families with additional functionality, as EC numbers were not assigned to KEGG annotations for those proteins in the reference genome. In addition to GH annotated proteins, other plant cell wall degrading proteins that target other compounds in degrading woody tissue such as esterases, pectinases, carbohydrate binding proteins, and others were identified (Table 4).

**Table 3:** Glycoside hydrolase (GH) families detected in MudPIT analysis.

GH Family	Number of Proteins	Protein ID	Interpro ID (or description if unknown)	KEGG EC Number (if known)	Secreted?
Candidate GH	2	78, 80	Blast homology		Y
Candidate GH 39	1	156	Blast homology		Y
Candidate GH 39	1	170	IPR000293, IPR002860		Y
Candidate GH5	1	34	Blast homology		Y
Candidate GH7	1	44	GH 7 superfamily domain	3.2.1.58: Glucan 1,3-beta-glucosidase	N
Candidate GH9	1	109	Blast homology		Y
Candidate GH 55	1	5	Pectin lyase 3 domain		Y
Candidate retaining beta glucosidase	1	54	JGI User annotation		Y
BNR repeat	2	35, 23	IPR002860		Y
Fungal cellulose binding	1		IPR000254		
Glycoside hydrolase - starch binding	1	120	IPR002044		N
1	1	27	IPR001360		Y
3	8	2, 56, 57, 58, 95, 149, 189, 254	IPR001764, IPR002772	3.2.1.21: Beta glucosidase	5Y 3N
5	3	26, 51, 209	IPR000254, IPR001547, IPR001764, IPR002772		Y
6	1	20	IPR001524		Y
7	4	1, 96, 115, 170	IPR001722, IPR000254		Y
10	1	21	IPR001000		Y
11	1	145	IPR001137		Y
13	2	75, 318	IPR006046, IPR004193	3.2.1.1: Alpha amylase	1 Y 1N
15	1	6	IPR000165	3.2.1.3: Glucan 1,4-alpha-glucosidase	Y
16	2	16, 87	IPR000757	3.2.1.73: Lichenase	Y
17	1	237	IPR000490	3.2.1.58: Glucan 1,3-beta-glucosidase	N
20	1	11	IPR001540	3.2.1.52: Beta-N-acetylhexosaminidase	Y
24	1	50	IPR002196		Y
28	1	112	IPR001002, IPR001223, IPR011583	3.2.1.14: Chitinase	N
31	1	4	IPR000322	3.2.1.20: Alpha glucosidase	Y
32	1	98	IPR001362		Y
35	1	323	IPR001944	3.2.1.23: Beta galactosidase	Y
37	1	25	IPR001661	3.2.1.28: Alpha trehalase	Y
43	1	184	IPR006710		Y
45	1	52	IPR000254, IPR000334		Y
61	1	163	IPR005103		N

**Table 4:** Other cell wall degrading proteins from MudPIT analysis.

Protein Name	Number of Proteins	Protein ID	Interpro ID (or description if unknown)	KEGG EC Number (if known)	Secreted?
Alpha-L-arabinfuranosidase	2	9, 79	IPR010720, IPR007934		Y
Candidate β-N-acetylhexosaminidase	1	168	IPR002022		Y
Candidate carboxylesterase	1	292	IPR001087		Y
Candidate ester hydrolase	1	402	Blast homology		N
Candidate pectin lyase	1	122	Blast homology		Y
Carbohydrate binding protein	1	393	IPR002889		Y
Carboxylesterase	3	30, 77, 269	IPR000379, IPR000408, IPR002018	3.1.1.1: Carboxylesterase	2Y 1N
Chitin binding protein	1	287	IPR001002, IPR002889		Y
Chitin deacetylase	1	99	IPR009939		Y
Cutinase	2	73, 278	IPR000379, IPR000675, IPR011150		Y
Esterase	3	72, 108, 136	IPR000379, IPR007312, IPR008262		2Y 1N
Galactose epimerase	1	22	IPR008183	5.1.3.3: Aldose 1-epimerase	N
Lipolytic enzyme	2	153, 331	IPR001087		Y
Pectate lyase	1	116	IPR004898		Y
Polysaccharide deacetylase	1	134	IPR002509		Y
Tannase and feruloyl esterase	1	159	IPR011118		N

Several proteins that may have the ability to degrade components of lignin or disrupt the linkage between lignin and other components of lignocellulose were identified (Table 5). These include laccases, tyrosinases, radical copper oxidases, oxidoreductases, and superoxide dismutases that are often associated with lignin degradation. Also, a candidate cellobiose dehydrogenase was detected, which can generate hydroxy radicals. In addition, enzymes that target aromatic compounds for degradation were found, including an esterase and biphenol reductase (Table 5).

**Table 5:** Proteins associated with lignin metabolism from MudPIT analysis.

Protein name	Number of Proteins	Protein ID	Interpro ID (or description if unknown)	KEGG EC Number (if known)	Secreted?
Acid phosphatase	4	18, 68, 135, 267	IPR000120, IPR000560, IPR002828, IPR003778, IPR003833, IPR004843	3.1.3.2: Acid phosphatase	Y
Alkaline phosphatase	1	97	IPR001952	3.1.3.1: Alkaline phosphatase	Y
Candidate 2-hydroxy-6-oxo-6-pheylhexa-2,4 dienoate hydrolase	1	201	IPR000073, IPR000379, IPR003089, IPR008262		Y
Candidate cellobiose dehydrogenase	1	199	CBM and DOMO domain containing protein		Y
Candidate copper radical oxidase	1	138	IPR002889+Blast homology		Y
Candidate esterase directed at aromatic compounds	1	199	IPR000379		N
Candidate FAD oxidoreductase	1	404	KOG2852: possible oxidoreductase+Blast homology		N
Carboxymuconolactone decarboxylase	1	381	IPR003779		N
Catalase	2	40, 114	IPR002226, IPR002818, IPR010582	1.11.1.6: Catalase	1Y 1N
FAD oxidoreductase	5	128, 165, 211, 219, 247	IPR006094		4Y 1N
Glyoxylase dioxygenase	1	398	IPR002110, IPR011588		N
GMC oxidoreductase	2	85, 124	IPR000172, IPR007867	1.1.99.1: Choline dehydrogenase	Y
Laccase	4	33, 133, 154, 261	IPR001117, IPR002355, IPR006162	1.10.3.2: Laccase1.10.3.3: L-ascorbate oxidase	2Y 2N
Nickel superoxide dismutase	1	106	IPR006162	1.15.1.1: Nickel superoxide dismutase	Y
Small secreted protein	3	94, 144, 290	Blast homology		1Y 2N
Superoxide dismutase	2	47, 248	IPR001189, IPR001424	1.15.1.1: Superoxide dismutase	N
Tyrosinase	2	37, 285	IPR002227		1Y 1N

In addition, 47 proteins relevant to protein digestion and nitrogen scavenging were detected in our protein extract, which were classified to 24 different IPR protein domains (Table 6). The most abundant domains involved in protease activity were IPR000209 (peptidase S8 and S53, subtilisin, kexin, sedolisin), IPR007484 (peptidase M28), IPR000834 (peptidase M14), IPR001461 (peptidase A1), and IPR 001563 (peptidase S 10 serine carboxypeptidase). Through cross annotation with the KEGG database, we classified proteins containing these domains to enzyme classes: proteins classified as IPR000209 were assigned to ECs 3.4.21.-(serine endopeptidases), 3.4.21.48 (cerevisin), and 3.4.14.9(tripeptidyl-peptidase I); proteins classified as IPR007487 were assigned to ECs 3.4.11.- (aminopeptidase), 3.4.11.10 (bacterial leucylaminopeptidase); proteins classified to IPR000834 were assigned to ECs 3.4.17.- (metallocarboxypeptidase), 3.4.17.15 (carboxypeptidase A2), 3.4.17.2 (carboxypeptidase B); proteins classified to IPR001461 were assigned to ECs 3.4.23.1 (pepsin), 3.4.23.24 (*Candida* pepsin), 3.4.23.-(aspartic endopeptidases); and proteins classified as IPR001563 were assigned to ECs 3.4.16.6 (carboxypeptidase D). Two proteins relevant to nitrogen recycling and nitrogen scavenging were also detected, including IPR03778 (urea amidolyase related) and IPR004304 (acetamidase/formamidase) respectively. Although IPR003778 could not be annotated with KEGG, IPR004304 was classified as EC 3.5.1.49 (formamidase).

**Table 6:** Proteinases and nitrogen recycling proteins identified from MudPIT data.

Protein name	Number of Proteins	Protein ID	Interpro ID (or description if unknown)	KEGG EC Number (if known)	Secreted?
Amidase	1	29	IPR000120		Y
Amidohydrolase	1	307	IPR006680		Y
Aminopeptidase	4	83, 88, 118, 256	IPR003137, IPR007484	3.4.11.-: Aminopeptidase	Y
Aspartic endopeptidase	2	102, 104	IPR000250, IPR001461 IPR001969	3.4.23.-: Aspartic endopeptidase	Y
Bacterial leucyl peptidase	1	63	IPR007484	3.4.11.10: Bacterial leucyl aminopeptidase	Y
Candidapepsin	1	24	IPR001461	3.4.23.24: Candidapepsin	Y
Candidate formylmethionine deformylase	1	322	Blast homology		N
Carboxypeptidase A	1	224	IPR000834		Y
Carboxypeptidase A2	1	64	IPR001412	3.4.17.15: Carboxypeptidase A2	Y
Carboxypeptidase B	1	266	IPR000834	3.4.17.2: Carboxypeptidase B	Y
Carboxypeptidase C	2	152	IPR000379, IPR001563	3.4.16.5: Carboxypeptidase C	Y
Cerevisin	2	43, 131	IPR000209, IPR003137	3.4.21.48: Cerevisin	Y
Cysteine peptidase	1	178	IPR000169		N
Di- and tri- peptidyl peptidase	1	117	IPR000379, IPR001375, IPR002088	3.4.14.-: Dipeptidyl peptidase and tripeptidyl peptidase	N
Formamidase	1	212	IPR002469, IPR004304	3.5.1.49: Formamidase	N
Fungalysin	1	55	IPR001842, IPR006025, IPR011096		Y
Glutamate carboxypeptidase II	1	90	IPR003137, IPR007365, IPR007484	3.4.17.21: Glutamate carboxypeptidase II	N
Metallocarboxypeptidase	1	62	IPR000834	3.4.17.-: Metallocarboxypeptidase	Y
Metalloendopeptidase	2	250 260	IPR001384, IPR001567, IPR006025		1Y 1N
Pepsin A	2	14, 380	IPR001461, IPR001969	3.4.23.1: Pepsin A	Y
Peptidase	1	13	IPR000379, IPR008758	3.4.-.-: Acting on peptide bonds (peptide hydrolases).	Y
Peptidase C2	1	171	IPR001300		N
Serine carboxypeptidase D	2	10	IPR001563	3.4.16.6: Carboxypeptidase D	Y
Serine endopeptidase	4	17, 49, 277, 396	IPR000209, IPR010259	3.4.21.-: Serine endopeptidase	Y
Tri-peptidyl peptidase	2	82, 221	IPR000209	3.4.14.9: Tripeptidyl-peptidase I	1Y 1N
Trypsin	1	46	IPR001314, IPR008256	3.4.21.4: Trypsin	Y
Urease	1	164	IPR000089, IPR000120, IPR003778, IPR003833		N

**Figure 1:**
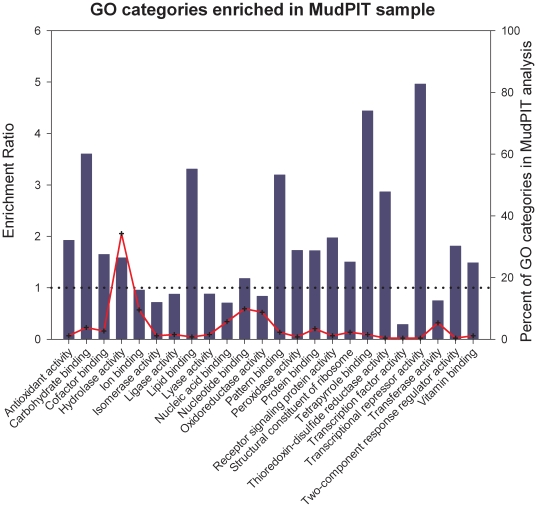
Enrichment of GO Molecular Function terms in proteomic analysis. Bar graph represents the ratio of % composition of term in proteomic data vs. % composition in the genome annotation. Values over 1 (dotted line) are overrepresented in the proteomic data. Red line illustrates the relative abundance of the GO term in the proteomic data.

Gene ontology (GO) annotation can be utilized to classify proteins by general function and overabundance of certain categories in expression datasets, including transcriptomes and proteomes, which can indicate the potential importance of these groups of genes to ongoing metabolic processes. In the proteome data obtained from an *A. glabripennis* -derived *F. solani* isolate growing on solid wood substrate, the most highly abundant categories from level 3 of the Molecular Function category were hydrolase activity, nucleotide binding, and oxidoreductase activity (red line, Figure 1), accounting for 34.2%, 9.9%, and 8.7% of proteins in our secretome, respectively. However, it is likely that these GO categories may be overrepresented in the reference genome and the overabundance of these categories in our proteome may simply be an artifact of genome enrichment. To correct for this and to identify GO categories of proteins that are truly overrepresented under these growth conditions, we compared the relative abundances of GO categories in our proteome against their relative abundances in the genome. Through this comparison, many GO categories are enriched in our proteome relative to the reference genome, including carbohydrate binding, hydrolase activity, peroxidase activity, and protein binding (bar graph, Figure 1).

### Verification of enzyme activity through *in vitro* lignocellulase activity

The fungal enzyme extract created from a solid wood culture of *A. glabripennis*-derived *F. solani* was surveyed for activities characteristic of enzymes that can degrade lignin, cellulose, and xylan (Table 7). β-glucosidase activity, measured by the release of reducing sugars from salicin, was 212.8 U/ml, CMCase activity was 13.1 U/ml, and cellulase activity measured by release of reducing sugar from microcrystalline cellulose (Avicel), was 14.7 U/ml (Table 7). Xylanase activity, measured by release of reducing sugar from birch wood xylan, was 70.5 U/ml. Although the sample did not exhibit lignin peroxidase activity as measured by oxidation of veratryl alcohol, the sample exhibited low levels of Mn-dependent peroxidases activity (0.021 U/ml), Mn-independent peroxidase activity (0.47 U/ml) and laccase activity (0.42 U/ml) (Table 7).

**Table 7:** Verification of lignocellulytic activity of *A. glabripennis* derived *F. solani* solid wood culture extracts through in vitro assays.

	Enzyme Activity (U/ml)*	Protein Conc. (mg/ml extract)	Specific Activity (U/mg protein)*
Cellulose/xylan			
Beta-glucosidase	12.77	0.06	212.8
CMCase	0.79	0.06	13.1
Cellulase (from Avicel)	0.88	0.06	14.7
Xylanase	4.23	0.06	70.5
Lignin			
Lignin Peroxidase	0	0.05	0
Mn-dependent Peroxidase	0.021	0.05	0.42
Mn-independent Peroxidase	0.47	0.05	9.4
Laccase	0.42	0.05	8.4

Activities of cellulases, xylanases, and lignin peroxidases, Mn-dependent and independent peroxidases, as well as laccase were measured for *F. solani* extracts with each assay containing 50 µg of protein.

* 1 unit of activity = amount of enzyme that releases 1 µmol of reducing sugar per minute for reducing sugar assays and amount of enzyme that oxidizes 1 µmol substrate per min for peroxidase assays.

### Verification of enzyme presence and activity through PAGE gel analysis

**Figure 2:**
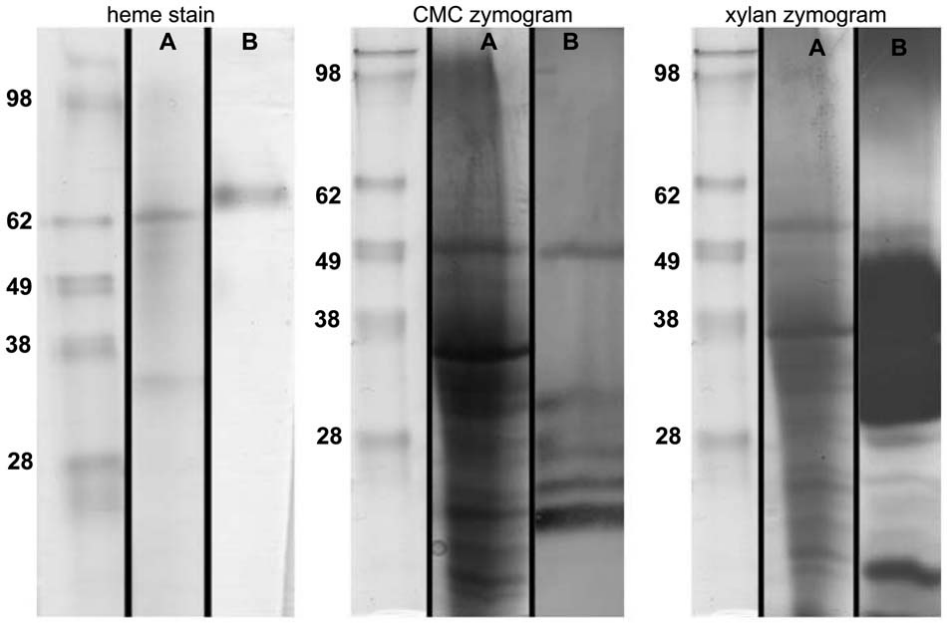
Heme staining and zymogram analysis of *A. glabripennis* derived *F. solani* solid wood culture extract. Twenty µg of fungal extract were loaded into each lane. Protein standard is on the left, with band sizes labeled (kDa). Lane A is a colloidal blue stained lane, and lane B is the corresponding heme stain/CMC/or xylan zymogram lane. For heme stain, a single band is present at approximately 70 kDa. For CMC zymogram six major zones of clearing are present at approximately 55, 32, 30, 27, 23 and 20 kDa and can be matched to protein bands on the colloidal blue stained lane. For birch wood xylan zymogram, many zones of clearing are present on the gel with major spots between 30 and 50 kDa. These correspond to a broad range of bands on the colloidal blue stained lane.

In addition to verification of activity in *in vitro* assays, PAGE analysis was performed to visualize active enzymes. A single heme containing protein of approximately 70 kDa was detected on a native heme stained gel; however, a corresponding protein band was not detected on the reference colloidal blue stained gel (Figure 2). A possible explanation for not observing a matching protein on the reference gel is that this protein may not be highly abundant or concentrated enough to be adequately visualized with the colloidal blue stain. Through zymogram analysis, carboxymethyl cellulase activity was detected and six major zones of clearing ranging from 20 to 55 kDa were identified (Figure 2). Comparison to the reference colloidal blue stained SDS-PAGE gel revealed that several similarly sized protein bands were present, including a major band at 55 kDa and several less intense bands around and below 28 kDa. Relatively high xylanase activity was also detected through birch wood xylan zymogram analysis; many zones of clearing were observed ranging in size from 20 kDa to 50 kDa (Figure 2). Very large and broad zones of clearing between 25 and 45 kDa obstructed the ability to define specific protein bands with activity towards xylan, but distinct bands can be seen above and below this region on the zymogram gel. Again, these regions of clearing can be matched to corresponding protein bands on the reference colloidal blue stained gel. This analysis verifies the data from *in vitro* analysis and also demonstrates that many of these activities are from a diversity of proteins, which is also represented in the proteomic dataset.

## Discussion

Over 30 years of research has been devoted to resolving the mechanisms and enzymology of lignin biodegradation, yet only three fungal enzymes have been discovered that conclusively depolymerize lignin, fully converting it to carbon dioxide and water. Reasons commonly cited for lack of progress in this field include a poorly resolved biochemical structure of lignin, unreliable *in vitro* assays to confirm lignin degrading activities, and uncharacterized growth and physiological conditions required for induction of lignin degrading enzymes in non-white rot fungal isolates. While advances in next-generation sequencing and bioinformatics may allow us to detect lignin, manganese-, and versatile- peroxidase orthologs in the genomes of newly sequenced organisms, validating and confirming that these orthologs actually catalyze lignin depolymerization is not a trivial task. Despite these difficulties, lignin degradation has recently been documented in the guts of two evolutionarily distant insect species: *Anoplophora glabripennis* and *Zootermopsis angusticollis* (Pacific dampwood termite) [12], [17], [20], [22], [24], [31], [32], [33], [34]. Since this initial discovery, much effort has been devoted to dissecting lignin degradation mechanisms and mining for key enzymes linked to these processes in both termites and *A. glabripennis*; however, the fundamental enzymes involved that catalyze these reactions remain elusive. Despite this ambiguity, there is evidence that all major reactions associated with large scale lignin biodegradation occur in both insects [22], but the predominant oxidative reactions are different. For example, side chain oxidation was the dominant reaction in the *A. glabripennis* gut, while ring hydroxylation was the dominant reaction in *Zootermopsis*, indicating that different enzymes may catalyze lignin degradation in these systems [22]. Despite these differences, one resounding commonality can be noted: these processes both occur in the absence of white rot basidiomycete fungi, leading to the possibility that novel lignin degrading enzymes are harbored in the insect genomes (not likely) or within the gut communities (more likely). While the gut bacterial communities of many termites and *A. glabripennis* are both dominated by Actinomycete bacteria [23], [64], including taxa known to efficiently metabolize aromatic compounds, the ability of these actinomycetes to completely depolymerize the lignin macromolecule remains questionable [17], [18], [19], [20], [21]. In addition, little is known about the composition of fungal communities in the guts of non-fungus cultivating termites; while in contrast, *A. glabripennis* larvae consistently harbor a filamentous ascomycete belonging to the *Fusarium solani* species complex [35], a metabolically diverse group of fungi with prolific lignin and aromatic polymer degrading capabilities [40], [46]. Here, we investigate the ability of this *A. glabripennis* -derived *F. solani* isolate to colonize and thrive on a solid wood substrate, degrade intractable woody polymers, including cellulose, xylan, and lignin, and extract other essential nutrients from this environment during periods of secondary metabolism.

In white rot basidiomycetes, expression of lignin degrading genes occurs solely during periods of secondary metabolism and nutrient limitation [10]; however, the conditions required for induction of lignin degrading enzymes are not well characterized in *Fusarium spp.* and their ability to colonize lignocellulose-based substrates varies tremendously [50]. Our goals to ensure substrate colonization regardless of inherent metabolic potential and to induce growth conditions characteristic of secondary metabolism were achieved. Proteins detected in MudPIT data associated with secondary metabolism include cupin-, germin-, and patatin-containing proteins, which facilitate nutrient storage under nutrient limiting conditions [65] (Table S6)). Additionally, proteins and enzymes typically induced during periods of stress and nutrient deprivation were detected including cell wall spore proteins, cytochrome p450, extracellular ribonucleases, gamma glutamyl transpeptidase, heat shock proteins, mucin-like glycoproteins, and woronin body proteins [65], [66], [67] (Table S6), likely indicating that this fungus was not persisting under nutrient rich, stress free conditions in culture. Furthermore, proteins associated with host plant interactions were detected from MudPIT data, including several candidate toxin-producing proteins and one cerato-platanin necrosis-inducing enzyme (Table S7) [68], indicating that the fungal isolate was not simply persisting on millet and bran and was actively colonizing wood chips present in the medium.

Likewise, cellulase, glycoside hydrolase (directed at β-1,4 linkages), and xylanase activities were conclusively detected *in vitro* through zymogram analysis and reducing sugar assays (Figure 2 and Table 7), demonstrating that this isolate was actively digesting carbohydrate polysaccharides present in the woody substrate rather than simply extracting glucose from the soluble starches present in millet. In tandem, GH families responsible for hydrolyzing β-1,4 linkages present in both cellulose and xylan were identified through MudPIT analysis, including complete enzyme complexes for full conversion of both polysaccharides to glucose. Cellulose alone requires at least three distinct enzymes for efficient glucose liberation, including endoglucases, exoglucanases, and β-glucosidases. Endoglucanases cleave amorphous sites in cellulose at random, decreasing its crystallinity, increasing its solubility, and rapidly exposing reducing and non-reducing ends to other hydrolytic enzymes. Subsequently, exoglucanases act on these reducing and non-reducing termini to release cellobiose and other cello-oligomers, which can be efficiently converted to glucose by β-glucosidases [69]. Endoglucanases (EC 3.2.1.4) are categorized into several GH families, including GH 5, 6, 7, 8, 9, 12, 44, 45, 48, 51, and 61. Exoglucanases or cellobiohydrolases classified into two distinct KEGG ECs depending on whether they target reducing or nonreducing ends and whether they have inverting or retaining activities: EC 3.2.1.176 (GH7 and 48; reducing, retaining), EC 3.2.1.91 (GH 5, 6, and 9; non-reducing, inverting). β-glucosidases (EC 3.2.1.21) are distributed among GH families 1, 3, 9, 30, 116 [70]. GHs with potential relevance to cellulose digestion detected through MudPIT analysis include GHs belonging to families 1, 3, 5, 6, 7, 45, and 61. Many of these glycoside hydrolases were not well-annotated in the reference genome with GO or KEGG terms [48], so the precise reactions catalyzed by these GHs cannot be directly inferred; an individual GH family can harbor enzymes with very diverse catalytic and substrate specificities (Table 3). However, the distribution of GHs in conjunction with release of reducing ends from microcrystalline cellulose, carboxymethyl cellulose, and salicin suggests that this fungal isolate possesses the full suite of cellulolytic enzymes.

On the other hand, hemicellulose is a much more heterogeneous polymer containing many monomeric subunits and greater diversity of chemical linkages, and thus, requires a combination of glycoside hydrolases and esterase enzymes for efficient conversion to sugar monomers [9]. For example, O-acetylglucuronoxylan is the predominant polysaccharide in hardwood trees [71] and requires endo-, exo-, and β-xylosidases (EC 3.2.1.37: GH 3, 30, 39, 43, 52, 54, 116, and 120) [70] to hydrolyze β-1,4 linkages; α-uronidsases (EC 3.2.1.139) to liberate glucuronic, mannuronic, and galacturonic acids; and ferulic acid esterase (EC 3.1.1.73) and acetylxylan esterase (EC 3.2.1.72) [34], [72] to hydrolyze phenolic ester bonds that cross-link hemicellulose to lignin. Of the eight GHs families with documented involvement in xlyan degradation, three were detected in our proteome that could account for the xylanase activity we observed *in vitro* in reducing sugar assays and zymogram analyses (Table 7 and Figure 2). These include GH families 3, 43, and a candidate GH 39 (Table 3). Many of these had predicted signal peptides, but no specific GO or EC annotations were present in the reference genome that could be utilized to determine substrate specificities. In addition, a number of ester hydrolyzing enzymes, including carboxylesterases, esterases, and a tannase/feruloyl esterase, were also detected through shotgun proteomics (Table 4). Surprisingly, a number of proteins with activity directed at pectin and cutin polymers, which are not highly abundant in woody tissues, were also detected; however, pectin and cutin are highly pertinent to wood decay processes because these compounds are often found in the central location of pit membranes, ray cell walls, and middle lamellae of wood cell walls [73] (Table 4). These polysaccharides are often broken down during wood rot processes and decomposition is often coupled to metal cation acquisition as pectin serves as a calcium reservoir in woody tissue [74].

Proteins are also occasionally found impregnating xylem elements and plant cell walls in woody tissue, which provide vital nitrogen sources that could be assimilated by wood degrading bacteria and fungi. Only a few types of proteins are co-localized to cell walls in wood and they are often covalently cross-linked to cellulose, hemicellulose, and lignin in the cell wall matrix [11]. Incidentally, extracellular proteinases are often highly expressed in white rot basidiomycetes during periods of active lignin metabolism [16]. As production of lignin degrading peroxidases is induced by nitrogen limiting conditions in white rot basidiomycetes, many have hypothesized that these fungi may degrade lignin in order to access proteins cross-linked to lignin, though this has not been directly tested [72], [74], [75]. In concert, we detected many extracellular proteinases in our own secretome, including many enzymes with broad substrate specificities that could serve to scavenge nitrogen from woody tissue, including aspartic peptidases, carboxypeptidases, metallopeptidases, and serine peptidases (Table 6). An alternative explanation for the abundance of proteinases in the secretome is that they may have been actively hydrolyzing proteins present in the millet, although these elements should have been depleted from the medium at the time of harvesting. In addition, fungi that thrive under these nitrogen limiting conditions for extended periods of time may also efficiently recycle and reuse nitrogenous waste products in amino acids and nucleotides. Two putative nitrogen recycling proteins were also detected, including formamidase and urease that actively convert nitrogenous waste into ammonia, which can subsequently be re-integrated into amino acids or nucleotides (Table 6) [76].

Protein, cellulose, and hemicellulose in wood chips are all protected from hydrolytic enzymes by lignin, a structural biopolymer dominated by recalcitrant linkages that can only be broken through radical oxidative depolymerization catalyzed by lignin-, manganese-, and versatile- peroxidases [10]. In addition, highly reactive hydroxyl radicals produced from Fenton reactions may help to expedite complete lignin depolymerization in some white rot fungi [50], [77]. While these enzymes all catalyze lignin depolymerization using slightly different mechanisms, they all require extracellular peroxide, which is generated by variety of enzymes including FAD oxidases, copper radical oxidases, glyoxal oxidases, and GMC oxidoreductases [10], [16], [77], [78]. Some lignin degrading fungi also utilize laccases [79] to catalyze oxidative cleavage of phenoxy linkages, which may degrade small lignin metabolites released by larger scale depolymerization processes or augment large scale oxidative depolymerization of lignin-, manganese-, or versatile- peroxidases. Although their catalytic potential can be expanded to oxidize more recalcitrant linkages in the presence of synthetic mediators [15], no natural varieties of these redox mediators have been conclusively identified, though some speculate that small secreted proteins expressed during active lignin metabolism may fill this niche [16].

Despite the presence of a lignin peroxidase ortholog in our reference genome, no *bona fide* lignin peroxidases or manganese peroxidases were detected through *in vitro* biochemical assays or *de novo* peptide sequencing; however, one unidentifiable heme protein was detected (Figure 2). Whether or not this protein functions as an extracellular lignin peroxidase is unknown. Additionally, many extracellular enzymes typically observed in the secretomes of other lignin-degrading fungi were detected, including several secreted laccases whose activity was verified *in vitro* (Tables 6 and 7) and two intracellular polyphenol oxidases (tyrosinases). Both phenol oxidases can oxidize similar substrates, but only laccases can oxidize syringaldazine, while trysoninases are more instrumental in degrading tannic and gallic acids [43], [80], [81]. In tandem, several extracellular peroxide generating enzymes, including FAD oxidases, GMC oxidoreductases, a putative copper radical oxidase, and superoxide dismutases, and one hydroxy radical generating enzyme (candidate cellobiose dehydrogenase) were also observed (Table 6), suggesting that processes requiring peroxide and hydroxyl radicals were occurring in the extracellular environment. In addition, several germin proteins were detected, which can also function as oxalate oxidases (Table S8). Oxalate is often produced during periods of active lignin metabolism and can be directly converted to hydrogen peroxide by oxalate oxidase and often enhance oxidative activities of manganese peroxidase *in vitro* [82]. Several enzymes that degrade small aromatic compounds were also identified, including a candidate reductase with activity directed at biphenyl compounds and a candidate esterase with activity directed at aromatic compounds [83], [84], which could hydrolyze bonds in small lignin metabolites produced from larger-scale biodegradation processes (Table 6). Finally, several small secreted proteins and hypothetical proteins were also detected in the culture supernatant; whether or not these proteins could be relevant to lignin degradation is unknown (Table 6 and Table S8).

Although no lignin peroxidase activity was detected *in vitro*, this does not necessarily indicate that this isolate does not harbor lignin degrading enzymes or possess lignin degrading capabilities. In fact, veratryl alcohol oxidation is often not detected during active lignin degradation in white rot fungi in extracellular extracts prepared from isolates growing on woody substrates, even when lignin peroxidase isozymes were detected in 2D gels or by *de novo* peptide sequencing. Furthermore, extensive glycosylation or other post translational modifications can interfere with trypsin digestions or alter protein masses, resulting in inefficient digestion or errors in predicted amino acid sequences, which can result in protein non-detection [72], [75]. It is also possible that the lignin peroxidase ortholog detected in the *Nectria haematococca* reference genome is not present in our isolate. An alternate explanation is that perhaps laccases are more involved in natural lignin degradation in this system than originally demonstrated. Lending support to this speculation is the observation that the lignin degrading capacity is strongly reduced in some laccase-deficient *Pycnoporus cinnabarinus* and *Sporotrichum pulverulentum* mutants and the hypothesis that small secreted proteins produced during active lignin metabolism may serve as natural redox mediators for laccase, enhancing its oxidative potential [16], [85], [86]. In addition, Scharf and colleagues [34] recently discovered an endogenous termite laccase whose phenol oxidase activity is enhanced in the presence of hydrogen peroxide, a characteristic more synonymous with lignin peroxidases. While this could indicate that this laccase has an inherently higher redox potential and can catalyze oxidation of more recalcitrant linkages than previously characterized laccases, this observation could also be an artifact of His-labeling and future studies are needed to validate its redox potential and determine its catalytic capabilities.

In conclusion, we have demonstrated that this *F. solani* isolate has definitive abilities to degrade proteins, cellulose, hemicellulose, and other carbohydrate polymers present in woody tissue and that it expresses many enzymes that are often up-regulated during periods of lignin metabolism in other lignin degrading fungi, including enzymes involved in extracellular peroxidase generation, laccases, and polyphenol oxidases. While we have not definitively documented full lignin depolymerization elicited by this *A. glabripennis* -derived *F. solani* strain, these results indicate that this isolate may have lignin degrading potential. In order to better assess the true metabolic potential of this fungal strain, whole genome sequencing is currently in progress to produce a more suitable reference genome for future transcriptomics and proteomics studies and more conclusively assay lignin degrading capabilities. Furthermore, follow up metatranscriptomic and metaproteomic approaches will be employed to determine if fungal transcripts and enzymes are actively expressed in the gut and to assess its potential contribution to lignin degrading activities in larval *A. glabripennis*.

## Supporting Information

Table S1 **Proteins identified in MudPIT analysis, with Necha 2.0 genomic database protein accession number, gene name, and protein score statistics from ProteinPilot software.**
(XLS)

Table S2 **Interpro annotation of proteins identified in MudPIT analysis.**
(XLS)

Table S3 **KEGG annotations of proteins identified in MudPIT analysis.**
(XLS)

Table S4 **Gene Ontology (GO) annotations of proteins identified in MudPIT analysis.**
(XLS)

Table S5 **SignalP signal peptide prediction of proteins identified in MudPIT analysis.**
(XLS)

Table S6 **Starvation and stress-related proteins from MudPIT analysis.**
(XLS)

Table S7 **Proteins related to Host plant interaction from MudPIT analysis.**
(XLS)

Table S8 **Hypothetical proteins detected from MudPIT data.**
(XLS)
